# Summary of the findings of the International Collaboration on Mild Traumatic Brain Injury Prognosis

**DOI:** 10.1186/s12998-014-0038-3

**Published:** 2014-11-04

**Authors:** James Donovan, Carol Cancelliere, J David Cassidy

**Affiliations:** Division of Health Care and Outcomes Research, Toronto Western Research Institute, University Health Network, University of Toronto, Toronto, Canada; Institute of Health Policy, Management and Evaluation, University of Toronto, Toronto, Canada; Institute of Sports Science and Clinical Biomechanics, Faculty of Health, University of Southern Denmark, Odense, Denmark

**Keywords:** Prognosis, Concussion, Mild traumatic brain injury

## Abstract

In 2004, the WHO Collaborating Centre for Neurotrauma, Prevention, Management and Rehabilitation Task Force published the first large systematic review and best evidence synthesis on the clinical course and prognosis for recovery after MTBI. Ten years later, the International Collaboration on Mild Traumatic Brain Injury Prognosis (ICoMP) formed to update the original WHO Task Force results. This summary review highlights important clinical findings from the full ICoMP results including the current evidence on the course and prognosis of recovery after MTBI in diverse patient populations (e.g., adults, athletes and children) and injury environments (e.g., motor vehicle collisions) as well as on the risk of long-term outcomes after MTBI, such as Parkinson’s disease and dementia. Additional clinical areas of interest in MTBI are also discussed including the similarities between MTBI and other traumatic injuries and the risk of Second Impact Syndrome after sport concussion. Clinicians can use this information to help inform patients on the likely course of recovery after MTBI/concussion and guide better decision-making in the care of these patients.

## Introduction

Over the past decade, mild traumatic brain injury (MTBI) or concussion has become a prominent public health concern. The annual incidence likely now exceeds 600 per 100,000 person-years and though popularized by injuries to prominent sports figures, MTBI most commonly occurs after falls and motor vehicle collisions [1]. In 2004, the WHO Collaborating Centre for Neurotrauma, Prevention, Management and Rehabilitation Task Force (WHO Task Force) published the first large systematic review and best evidence synthesis on MTBI course and prognosis. After reviewing the literature from 1980–2000, the WHO Task Force found the prognostic literature to be of poor quality [2]. For instance, inconsistent MTBI definitions, weak study designs and pervasive biases were common problems throughout the literature. In addition, knowledge gaps were found in such areas as prognosis in the elderly, risk of long-term outcomes, and non-surgical intervention studies [2]. The WHO Task Force concluded that more high quality prognostic research was needed to address these evidence gaps and advance the understanding on the course and prognosis after MTBI [3].

In 2011 an international group of 21 research scientists and clinicians, the International Collaboration on MTBI Prognosis (ICoMP), was formed and funded to update the WHO Task Force findings on MTBI prognosis (see Table 1) [4]. ICoMP undertook a systematic review and best-evidence synthesis to identify the clinical course and prognostic factors for recovery after MTBI within diverse patient populations (e.g., children, adults, athletes, and military personnel) as well as to provide insight into the risk of long-term outcomes, such as movement- and dementia-related disorders.

**Table 1:** **The complete list of all 21 ICoMP team members**

**ICoMP members**	**Institutional Associations**
J. David Cassidy, DC, PhD, DrMedSc	• Senior Scientist, Division of Health Care and Outcomes Research, Toronto Western Research Institute, University Health Network, University of Toronto, Canada.
	• Professor, Institute of Health Policy, Management and Evaluation, University of Toronto, Canada.
	• Professor, Division of Epidemiology, Dalla Lana School of Public Health, University of Toronto, Canada.
	• Globalization Professor, Institute of Sports Science and Clinical Biomechanics, Faculty of Health, University of Southern Denmark, Odense, Denmark
Linda Carroll, PhD	• Professor, Department of Public Health Sciences and Alberta Centre for Injury Control and Research, University of Alberta, Edmonton, Canada
Lena Holm, DrMedSc	• Associate Professor, Division of Epidemiology, Institute of Environmental Medicine, Karolinska Institutet, Stockholm, Sweden
Jörgen Borg MD, PhD	• Department of Clinical Sciences, Rehabilitation Medicine, Karolinska Institutet, Danderyd University Hospital, Stockholm, Sweden
Jan Hartvigsen DC, PhD	• Professor, Institute of Sports Science and Clinical Biomechanics, University of Southern Denmark, Odense, Denmark
	• Nordic Institute of Chiropractic and Clinical Biomechanics, Odense, Denmark
Pierre Côté DC, PhD	• Associate Professor, Faculty of Health Sciences, University of Ontario Institute of Technology, Oshawa, Canada.
	• UOIT-CMCC Centre for the Study of Disability Prevention and Rehabilitation, University of Ontario Institute of Technology, Toronto, Canada.
	• Associate Professor, Division of Epidemiology, Dalla Lana School of Public Health, University of Toronto, Canada.
	• Associate Professor, Institute of Health Policy, Management and Evaluation, University of Toronto, Canada.
Carol Cancelliere, DC, MPH	• Trainee, Division of Health Care and Outcomes Research, Toronto Western Research Institute, University Health Network, University of Toronto, Canada.
	• Doctoral Student, Institute of Health Policy, Management and Evaluation, University of Toronto, Canada.
Cesar Hincapié DC, MHSc	• Trainee, Division of Health Care and Outcomes Research, Toronto Western Research Institute, University Health Network, University of Toronto, Canada.
	• Doctoral student, Division of Epidemiology, Dalla Lana School of Public Health, University of Toronto, Canada
James Donovan DC	• Research Associate, Division of Health Care and Outcomes Research, Toronto Western Research Institute, University Health Network, University of Toronto, Canada
Victor Coronado MD, MPH	• Medical Epidemiologist, National Center for Injury Prevention and Control, Centers for Disease Control and Prevention (CDC), Atlanta, United States
Jean-Luc af Geijerstam MD, PhD	• Section Head, Department of Rehabilitation Medicine, Karolinska Institutet, Danderyd University Hospital, Stockholm, Sweden
Britt-Marie Stålnacke MD, PhD	• Adjunct Professor, Department of Rehabilitation Medicine, Community Medicine and Rehabilitation, Umeå University, Sweden
Connie Marras MD, PhD	• Assistant Professor, Division of Neurology, Faculty of Movement Disorder, University of Toronto
	• Morton and Gloria Shulman Movement Disorders Centre, and the Edmond J. Safra Program in Parkinson’s Research, Toronto Western Hospital, University Health Network, University of Toronto, Canada.
Vicki Kristman PhD	• Assistant Professor, Department of Health Sciences, Lakehead University, Thunder Bay, Canada
	• Institute for Work and Health, Toronto, Canada.
	• Division of Epidemiology, Dalla Lana School of Public Health, University of Toronto, Canada.
	• Division of Human Sciences, Northern Ontario School of Medicine, Lakehead University, Thunder Bay, Canada
Eleanor Boyle PhD	• Associate Professor, Institute of Sports Science and Clinical Biomechanics, Faculty of Health, University of Southern Denmark, Odense, Denmark.
	• Division of Epidemiology, Dalla Lana School of Public Health, University of Toronto, Canada
Michelle Keightly PhD	• Departments of Occupational Science and Occupational Therapy, Psychology and Graduate Department of Rehabilitation Science, University of Toronto, Canada.
	• Bloorview Research Institute, Holland Bloorview Kids Rehabilitation Hospital, Toronto, Canada.
L. Rachid Salmi MD, PhD	• Professor, University of Bordeaux, ISPED, Centre INSERM U897-Epidemiologie-Biostatistique, F-33000 Bordeaux, France (Salmi); INSERM, ISPED, Centre INSERM U897-Epidemiologie-Biostatistique, F-33000 Bordeaux, France (Salmi); CHU de Bordeaux, Pole de sante publique, Service d’information medicale, F-33000 Bordeaux, France
Ryan Hung, MD MSc	• Department of Rehabilitation and Complex Continuing Care, Holland Bloorview Kids Rehabilitation Hospital, Toronto, Canada.
	• Department of Pediatrics, University of Toronto, Canada
Alison Godbolt MBChB, MD,	• Department of Clinical Sciences, Rehabilitation Medicine, Karolinska Institutet, Danderyd University Hospital, Stockholm, Sweden
Catharina Nygren-de Boussard MD, PhD	• Department of Clinical Sciences, Rehabilitation Medicine, Karolinska Institutet, Danderyd University Hospital, Stockholm, Sweden
Peter Rumney MD	• Department of Rehabilitation and Complex Continuing Care, Holland Bloorview Kids Rehabilitation Hospital, Toronto, Canada

Information on the course and prognosis after MTBI can help clinicians make better informed decisions such as when a patient can safely return to work or play and it can also help clinicians identify those patients who may not be recovering as expected [3]. Further, it is important for clinicians to understand what factors determine prognosis in MTBI, especially those that are potentially modifiable. For example, the WHO Task Force found the presence of spine and head-related pain to be significant determinants of prognosis after MTBI [3]. Chiropractors as well as other health practitioners may be well positioned to improve care for MTBI patients by reducing pain-related MTBI symptoms and in turn may help play an important role in lessening the burden of this growing public health concern. The main purpose of this review is to present a summary of some of the key clinical findings of the full ICoMP results and to highlight other relevant clinical topics such as the similarities between MTBI and other traumatic injuries and the risk of Second Impact Syndrome after sport concussion.

## Review

### ICoMP search strategy and critical review process

ICoMP performed a comprehensive literature search from 2001–2012. All 10 included systematic reviews complied with the Preferred Reporting Items of Systematic Reviews and Meta-Analyses (PRISMA) statement [5]. Details of the full systematic search and review procedures are available in Cancelliere et al. [6]. Overall, ICoMP screened 77,914 records, reviewed 299 full-text articles, and deemed 101(34%) to be scientifically admissible, based on the Scottish Intercollegiate Guidelines Network (SIGN) criteria, and included in the ICoMP synthesis [7]. Key findings from papers found to be of low risk of bias were extracted into evidence tables. ICoMP undertook best-evidence syntheses by linking conclusions and recommendations to the evidence tables. Prognostic factor evidence was prioritized based on the phases of study framework described by Côté et al. [8]: Phase I studies are preliminary, hypothesis-generating investigations that explore crude associations between potential prognostic factors and disease outcomes; Phase II studies are exploratory and test associations between sets of prognostic factors and outcomes using multivariable analyses; Phase III studies are confirmatory analyses, including well-defined pre-stated hypotheses allowing for focused examination of the strength, direction and independence of a prognostic factor to disease outcome taking into account potential confounding factors. Phase III studies are confirmatory and provide the strongest prognostic evidence.

### ICoMP results

#### Sport concussion prognosis and return to play

By understanding sport concussion prognosis, clinicians can help to better educate athletes, coaches, and parents regarding their primary concerns, such as the likely duration of post-concussion symptoms, immediate versus delayed return-to-play (RTP), and potential sequelae of repetitive concussion. Similar to the results of the WHO Task Force, recent literature suggests that most athletes recover quickly (i.e., days to a few weeks) with respect to both cognitive functioning (e.g., attention and memory) and post-concussion symptoms (e.g., headache, fatigue, dizziness, etc.) [9]. While most athletes experience a favourable recovery, factors such as a history of previous concussion, number and duration of post-concussion symptoms and being a younger-aged/high school athlete are associated with delayed recovery. The literature also shows that cognitive functioning is not significantly (if at all) impaired and the mild deficits that do occur resolve quickly in high school, collegiate, and professional athletes [9].

Although RTP guidelines are popular there are no high-quality studies assessing their impact on recovery or prevention of additional injury and most of the research on RTP focuses on contact sports that include male professional American and Australian football players [9]. There is some evidence that the majority of athletes do RTP within the same game or within a few days afterward [9]. Despite the growing concern over RTP after sport concussion, research quality continues to be poor, demonstrating little to no methodological improvement since the WHO Task Force review. Furthermore, RTP decision-making tools, such as the Zurich Consensus guidelines, though widely used, continue to be based on expert opinion and clinical judgement, rather than scientific evidence [10].

In order to develop evidence-based recommendations for the management of sport concussion, ICoMP recommends randomized controlled trials to test the efficacy of existing RTP guidelines [9]. Sport concussion study methodologies remain highly heterogeneous (e.g., different follow-up durations and outcome measures), and include predominantly male American football players 16 years of age and older. Clinicians, however, are responsible for managing male and female athletes of all ages, skill levels, and sporting activities (e.g., hockey, soccer, basketball, etc.) and for these groups, evidence is lacking. Firm conclusions regarding the long-term outcome of repetitive sport concussion cannot be made at this time due to the lack of evidence. Conversely, short-term recovery after sport concussion was found to be delayed in athletes with a history of previous concussion and clinicians need to consider this when making future decisions about RTP or even ending sports participation, especially in younger-aged athletes and those at high risk for repeat concussion such as in contact sports.

One final concern in the sports setting is the potential for Second-Impact Syndrome (SIS) [11]. This very rare condition can occur when a second concussion or head injury happens before the brain has fully healed from a first concussion, resulting in increased intracranial pressure and death [11,12]. Some experts cite this concern when cautioning against early RTP [13]. The WHO Task Force found no scientific evidence on the incidence or risk of SIS, other than a few case reports from North America [1]. The WHO Task Force reviewed these cases and came to the conclusion that the evidence that a second concussion increases the risk for cerebral swelling and death is not well established and another systematic review [14] reached this same conclusion. ICoMP found no studies addressing SIS as an outcome after an initial concussion. While SIS may not be well established in the company of high quality scientific evidence, it nevertheless remains a potentially very serious complication after MTBI and as a result a real concern for clinicians and patients. Accordingly, there is an urgent need for improved research in the area of SIS, including better reporting of fatal outcomes after MTBI and better study designs to address the incidence and risk of SIS.

### Adult prognosis after MTBI

#### Self-reported symptoms

Self-reported symptoms (e.g., headache, fatigue, and dizziness) are the main reason for seeking care after MTBI. Indeed, ICoMP found adult self-reported symptoms to be the most frequently reported of outcomes after MTBI [15]. Recent evidence suggests that these symptoms are common and typically resolve over the course of weeks to several months [15]. Also, ICoMP found adult patients after MTBI may devalue or minimize pre-existing symptoms such as headache and fatigue and therefore misattribute symptoms occurring before the injury to the injury itself [15]. Furthermore, self-reported post-concussion symptoms are not unique or specific to head injury per se: injured control groups (i.e., orthopedic injuries) report similar post-traumatic symptoms. Since post-concussion symptoms appear both common and non-specific, the ICoMP recommends possibly dropping the diagnostic label of “post-concussion syndrome” and using the more appropriate “post-traumatic symptoms” as a replacement [15].

Despite post-concussion symptoms being common and resolving in the short-term for the majority, 22 to 36% of MTBI patients continue to report three or more post-traumatic symptoms six months after injury [15]. Interestingly, ICoMP found persistent symptom duration and overall recovery to be more associated with psychosocial factors, such as poor expectations for recovery and negative injury perceptions, rather than biomedical or injury-related factors such as duration of loss of consciousness (LOC) and post-traumatic amnesia (PTA), which are factors commonly used to diagnose and indicate initial head-injury severity.

The above results are in accordance with the WHO Task Force regarding the commonality and lack of specificity of adult self-reported cognitive and somatic symptoms [3]. However, symptoms can persist in a minority of patients and therefore may be challenging for the treating clinician. Clinical importance should be placed on early identification of injury perceptions and other psychosocial factors, as these appear to be strong determinants of recovery. Furthermore, much needed methods such as prognostic prediction rules could help clinicians to triage those at higher risk for poor recovery into early treatment programs, if such programs can be shown to be effective.

One important ICoMP accepted study developed a useful and easy-to-implement prediction rule identifying a favourable recovery six months after MTBI [16]. Prediction rules include combinations of prognostic factors, or markers, strongly associated with a particular outcome, favourable or otherwise. This study examined a range of pre-, peri- and post-injury prognostic factors and results demonstrated a 90% probability for favourable MTBI recovery in those who have no pre-existing physical problems, experience fewer and less severe post-concussion symptoms and suffer lower levels of post-traumatic stress. Absent from these results are the typical peri-injury biomedical factors that are often related to head-injury prognosis (e.g., LOC, PTA, and Glasgow Coma Scale (GCS)). In turn, this suggests the need to shift from a biomedical towards a more biopsychosocial model to understand MTBI recovery. ICoMP recommended that this clinical prediction rule be independently externally validated before being implemented in other settings [15].

#### Cognitive outcomes

Instead of relying on patient self-report, traditional or computerized neuropsychological testing is often used to more objectively measure deficits and monitor cognitive recovery after MTBI. The WHO Task Force results suggested that early cognitive deficits are common and usually resolve within one to three months after MTBI [3]. Similar to the WHO Task Force, recent evidence suggests adults commonly demonstrate cognitive deficits initially after MTBI, despite variability in the nature of these deficits (e.g., attention, distractibility, learning, etc.) [17]. ICoMP found substantial evidence for the presence of cognitive deficits in the first two weeks after injury, while the timing of recovery of these deficits is less well known. Although the WHO Task Force found good cognitive recovery within three months after MTBI, limited evidence now suggests for some, full recovery may take six months to a year [17].

#### MTBI after motor vehicle collisions (MVC)

It is important for clinicians to understand the course of recovery after MTBI within the context of different injury environments. For example, an ICoMP original study by Cassidy et al. [18] examined the incidence, course and potential prognostic factors in traffic-related MTBI in the Saskatchewan population. MTBI was found to affect 24% of those with traffic injuries, with a median time to recovery of 100 days; however, 23% of those affected continued to report not being recovered at one year. In those with MTBI, somatic symptoms such as neck pain (90%), headache (84%) and low- and mid-back pain (63%, 58%) were commonly reported. Back pain and headache were also found to be important factors associated with poor recovery in patients with MTBI. Moreover, strong determinants of MTBI recovery, in addition to pain, continue to be factors more psychosocial in nature (e.g., poor expectations of recovery, negative injury perceptions, etc.). This study reflects the reality of multiple injuries after traffic collisions and the fact that head injuries are often accompanied by neck injuries [19].

Another ICoMP original study by Hartvigsen et al. [20] used the same cohort as Cassidy et al. [18] to determine the symptom course and health-care use in MTBI sufferers after motor vehicle collision (MVC). Significant somatic pain complaints (e.g., headache and neck pain) were common, lasting up to one year after MTBI. Though less prevalent at one year, spine-related pain complaints such as neck and low back pain continued to be reported in up to one-quarter of subjects. Medical physicians provided care for the majority of patients (96%); however, many also sought early care from allied health professionals, with 42% seeing a physiotherapist and 20% seeing a chiropractor. For the majority with MTBI, care was provided by multiple health professionals, with non-medical physician care increasing over time. Furthermore, those most likely to report on-going symptoms also sought the most non-physician care. This study underlines the importance of somatic pain (e.g., head and spine pain) in the prognosis of MTBI.

Prognostic research on MTBI is now shedding light on possible similarities between MTBI and other traumatic injuries. For instance, similar post-traumatic symptom patterns can occur after whiplash, or MTBI including headache, neck pain, dizziness, fatigue, and concentration difficulties, to name a few [21]. Further, expectations for recovery appear highly prognostic for actual recovery in both MTBI and whiplash (i.e., those not expecting to get better tend to recover more slowly) [22,23]. In light of these similarities as well as others, it has recently been proposed that those suffering whiplash may also have also suffered a concussion and even more that direct impact to the head is no longer necessary for a diagnosis of concussion [13]. While similarities in post-traumatic symptom patterns and determinants of recovery do exist, care must be taken before blurring the diagnostic lines between MTBI and whiplash. Evidence-based definitions (e.g., WHO Task Force) include trauma to the head as an important diagnostic criteria for defining MTBI. However, it is possible that a coup-contrecoup mechanism of injury during whiplash might also negatively affect the brain. More research in this respect is required to fully delineate the relationship between MTBI and whiplash associated disorder (WAD).

#### Return to activity after MTBI

Current treatment methods for MTBI bear striking resemblance to historical methods once recommended for low back pain (LBP). For instance, a lengthy course of bed rest was once standard practice for acute LBP. However, this all changed after a randomized controlled trial showed that bed-rest prolongs LBP [24]. At present, the sport-medicine literature recommends full physical and cognitive rest until acute symptoms resolve as the universally accepted treatment after sport concussion, even though there are no trials showing this approach is the best way to manage MTBI [10]. Further, for adult patients, acute post-concussion symptoms such as headache, fatigue and concentration problems can last weeks to several months [15]; thus, recommending continued rest for these patients may not be prudent. Caution is also needed when recommending prolonged rest for patients with a low risk of repeat MTBI/concussion such as athletes not involved in contact sports and those injured after a fall or MVC. In a recent review, Silverberg & Iverson [25] caution against recommending full rest beyond three days for the majority who suffer MTBI, and suggest that management of MTBI be guided by specific patient circumstances and characteristics. Recommending prolonged rest might lead to de-conditioning and withdrawal from activities of daily living that could assist recovery. An ICoMP accepted randomized controlled trial showed that extensive rest was no better at improving recovery after MTBI than a briefer period of rest followed by a prompt return to normal activity [26]. Bear in mind, the current best evidence for LBP now discourages rest, having been replaced by ‘remain active’ as the new standard of care [27]. The ICoMP, in accordance with the WHO Task Force, also recommends early but careful activation for most MTBI patients [26]. However, the current authors recognize that clinical judgement in this respect is important.

#### Return to work (RTW)

MTBI can result from accidents occurring in various settings including the workplace and clinicians are often called upon to make a prognosis for return to work (RTW). The ICoMP performed the first systematic review and evidence synthesis on RTW prognosis after MTBI and found four admissible cohort studies [28]. The results indicate that most individuals RTW within three to six months. Predictors of full RTW after MTBI include high levels of education, younger age, absence of severe pain on initial hospital admission, job independence, and high job decision-making latitude [28]. Those factors not predictive however, include duration of LOC and PTA, dizziness, fatigue and headache. Injury-related factors have therefore only a partial impact on predicting RTW after MTBI, and this is consistent with studies of other injuries [29]. More evidence is needed to replicate these findings, and there is a need to investigate return to sustained employment (i.e., follow up beyond two years) in MTBI patients.

### Pediatric prognosis after MTBI

Parents and family members can have significant fears and concerns after a child suffers an MTBI. The fear that a child will suffer long-term physical or cognitive symptoms is certainly important, not to mention the eventual concerns over academic performance once the child returns to school. As a result, it can be challenging for clinicians to address parental and family worries while at the same time directing care for the injured child. The WHO Task Force review found the prognosis for children after MTBI to be good, with symptoms resolving within two to three months and little to no evidence of any lasting cognitive or academic deficits [3]. Recent evidence also suggests that for the majority of children, self-reported symptoms resolve over time and there are no MTBI-specific long-term cognitive deficits [30]. However, limited evidence from one Phase III and one Phase II study found that children with lower cognitive ability and a more complicated MTBI (i.e., intracranial MRI abnormalities) reported more post-concussion physical and cognitive symptoms at three months and one year post-injury, respectively [30]. Unfortunately, ICoMP did not find any acceptable studies on return to school after MTBI and highlight this as an area of priority for future research. Clinical practice guidelines on the management of pediatric MTBI including persistent symptoms have recently been published online and clinicians may wish to consult these guidelines when managing young, head-injured patients [31].

### Long-term outcomes after MTBI

There is increasing media attention and public concern over the purported risk that repeated MTBI can lead to subsequent chronic cognitive impairment (CCI), dementia, or to other distinct neurodegenerative disorders (e.g., chronic traumatic encephelopathy (CTE), Parkinson’s disease (PD)). Nearly a decade removed from the WHO Task Force, evidence addressing the possible latent outcomes after adult MTBI continues to remain insufficient: ICoMP found only one cohort study (Phase III) on dementia in adults after MTBI [32]. Limited evidence from this Phase III study indicates that a history of reported MTBI is not associated with a diagnosis of dementia, five years later [32]. ICoMP concluded that there is a lack of evidence of an increased risk of dementia after MTBI [32].

To scientifically answer the question “What is the risk of developing dementia or other neurodegenerative disorders after suffering repeat MTBI?” for example, requires cohort studies with long-term follow up, or more realistically, case–control studies. Unfortunately, studies to appropriately address these questions are still needed. The present literature is dominated by uncontrolled case reports and case series, such as the highly cited study by McKee et al. [33], which raise interesting hypotheses for example about CTE risk, but cannot infer causality.

ICoMP accepted five studies examining the risk of developing Parkinson’s disease (PD) following MTBI [34]. One Phase III study found an association between MTBI and PD, but only if the first PD diagnosis occurred within nine or less years of the initial injury. Further, no association was found when examining longer time delays (i.e., >10 years) between MTBI and PD development. Reverse causality however, likely explains the shorter time interval associated with injury and disease onset; that is to say that early stages of PD when it is not yet diagnosed may cause a fall resulting in MTBI. Collectively, the ICoMP evidence does not suggest an association between PD and MTBI [34].

In children however, there is limited evidence indicating possible long-term neurological sequelae after MTBI [30]. One ICoMP accepted Phase III study found children with MTBI to be twice as likely to develop epilepsy as those without [30]. The risk was highest in the first year after MTBI, but there remained a 50% higher risk of developing epilepsy even 10 years after injury. Although an elevated risk does exist, the absolute number of new cases of epilepsy in children after MTBI (i.e., the absolute risk) is actually quite low and this is important information to consider when addressing the concerns of parents, family members or others about this risk. For example, in children aged 5–10 years, this risk translated into 1.56 compared to 0.87 new cases of epilepsy per 1000 person-years in those with versus without a history of MTBI, respectively [30]. The previous WHO Task Force also found an increased risk of epilepsy in children within the first four years after MTBI [3]. Taken together, these consistent findings substantiate the small yet sustained risk of developing epilepsy after suffering MTBI as a child.

### Research recommendations

The ICoMP systematically searched and included 101 (34%) articles within the best-evidence synthesis on MTBI prognosis. This is virtually the same low acceptance rate reported by the WHO Task Force (i.e., 28%). Despite being a decade on from the original review, significant methodological issues persist in the MTBI literature [35]. For example, of those accepted studies, only 10% were Phase III confirmatory studies. In turn, MTBI prognosis research remains highly heterogeneous and primarily exploratory in nature (i.e., consists of mainly Phase I and II studies) [35]. Also, there is no universally accepted definition of MTBI and ICoMP tallied over 50 unique case definitions [35]. Significant knowledge gaps still remain. For instance, quality studies on prognosis after sport concussion fail to include female athletes, male and female athletes between 13–15 years of age, and sports other than American or Australian football. In addition, RTP guidelines have yet to be scientifically tested. Lastly, the current evidence largely comprises short-term studies with only one year of follow-up. As such, pertinent questions surrounding the long-term outcomes after MTBI remain unanswered.

## Conclusions

Despite the media’s attention, concussion or MTBI is not a problem limited only to professional athletes and those wanting to return-to-play. Children and adults suffer MTBI after motor vehicle collisions and falls and these patients all need to be informed on the likely course and prognosis of recovery after MTBI. In the acute management of MTBI patients, initial importance is placed on identifying the rare, but serious complications that can potentially occur after head injury, such as intracranial bleeding. Clinicians need to promptly identify those with new symptom onset or increasing symptom severity, as immediate neurosurgical attention may be required. While complications can occur, recovery is generally good for the majority after MTBI or concussion. Chiropractors as well as other clinicians can facilitate a path to good recovery for MTBI patients through early education and positive reassurance as well as by providing treatments aimed at reducing associated spine and headache-related pain. Similar to care for whiplash injuries and LBP, MTBI patients may benefit from early, but careful activation, while limiting passive care reliance. For those with persistent post-concussion symptoms, psychosocial factors (e.g., poor expectations of recovery, negative injury perceptions) as well as co-morbidities (e.g., somatic pain) need to be addressed and modified to help direct a patient’s course towards a positive recovery. Also for those with persistent symptoms, caution must be exercised before seeking excessive diagnostic testing or applying diagnostic labels (i.e., post-concussion syndrome) as unnecessary diagnoses as well as testing can increase patient anxiety and delay further recovery. Integrating care with a patient’s primary medical physician is recommended if additional symptom-specific treatments are required (e.g., pain medication or neurocognitive testing). Forming similar therapeutic partnerships may also be necessary to facilitate a plan for RTW after MTBI. While there is much work to be done to better understand MTBI prognosis, the recent ICoMP review has provided strong preliminary evidence which clinicians can use to help improve and inform clinical decision making and thus promote better recovery and limit disability after MTBI.

## Abbreviations

AD: Alzheimer’s disease
CCI: Chronic cognitive impairment
CDC: Centers for disease control
CTE: Chronic traumatic encephalopathy
GCS: Glasgow coma scale
ICoMP: International collaboration on MTBI prognosis
LBP: Low back pain
LOC: Loss of consciousness
MTBI: Mild traumatic brain injury
MVC: Motor vehicle collision
PD: Parkinson’s disease
PRISMA: Preferred reporting items of systematic reviews and meta-analyses
PTA: Post-traumatic amnesia
RTP: Return to play
RTW: Return to work
SIGN: Scottish intercollegiate guidelines network
SIS: Second impact syndrome
WAD: whiplash associated disorders
WHO: World Health Organization

## References

[1] Cassidy JD, Carroll LJ, Peloso PM, Borg J, von Holst H, Holm L, Kraus J, Coronado VG, WHO Collaborating Centre Task Force on MTBI (2004). Incidence, risk factors and prevention of mild traumatic brain injury: results of the WHO collaborating centre task force on mild traumatic brain injury. J Rehabil Med.

[2] Carroll LJ, Cassidy JD, Holm L, Kraus J, Coronado VG, WHO Collaborating Centre Task Force on MTBI (2004). Methodological issues and research recommendations for mild traumatic brain injury: the WHO collaborating centre task force on mild traumatic brain injury. J Rehabil Med.

[3] Carroll LJ, Cassidy JD, Peloso PM, Borg J, von Holst H, Holm L, Paniak C, Pepin M, WHO Collaborating Centre Task Force on MTBI (2004). Prognosis for mild traumatic brain injury: results of the WHO collaborating centre task force on mild traumatic brain injury. J Rehabil Med.

[4] Cancelliere C, Cassidy JD, Côté P, Hincapié CA, Hartvigsen J, Carroll LJ, Marras C, Boyle E, Kristman V, Hung R, Stålnacke BM, Rumney P, Coronado V, Holm LW, Borg J, Nygren-de Boussard C, Af Geijerstam JL, Keightley M (2012). Protocol for a systematic review of prognosis after mild traumatic brain injury: an update of the WHO collaborating centre task force findings. Syst Rev.

[5] Moher D, Liberati A, Tetzlaff J, Altman DG, PRISMA Group (2009). Preferred reporting items for systematic reviews and meta-analyses: the PRISMA statement. PLoS Med.

[6] Cancelliere C, Cassidy JD, Li A, Donovan J, Côté P, Hincapié CA (2014). Systematic search and review procedures: results of the International Collaboration on Mild Traumatic Brain Injury Prognosis. Arch Phys Med Rehabil.

[7] **Scottish intercollegiate guidelines network (SIGN).** In [http://www.sign.ac.uk/] 10.1177/003693300505000202 15977513

[8] Côté P, Cassidy JD, Carroll L, Frank JW, Bombardier C (2001). A systematic review of the prognosis of acute whiplash and a new conceptual framework to synthesize the literature. Spine (Phila Pa 1976).

[9] Cancelliere C, Hincapié CA, Keightley M, Godbolt AK, Côté P, Kristman VL, Stålnacke BM, Carroll LJ, Hung R, Borg J, Nygren-de Boussard C, Coronado VG, Donovan J, Cassidy JD (2014). Systematic review of prognosis and return to play after sport concussion: results of the International Collaboration on Mild Traumatic Brain Injury Prognosis. Arch Phys Med Rehabil.

[10] McCrory P, Meeuwisse WH, Aubry M, Cantu B, Dvorak J, Echemendia RJ, Engebretsen L, Johnston K, Kutcher JS, Raftery M, Sills A, Benson BW, Davis GA, Ellenbogen RG, Guskiewicz K, Herring SA, Iverson GL, Jordan BD, Kissick J, McCrea M, McIntosh AS, Maddocks D, Makdissi M, Purcell L, Putukian M, Schneider K, Tator CH, Turner M (2013). Consensus statement on concussion in sport: the 4th international conference on concussion in sport held in Zurich, November 2012. Br J Sports Med.

[11] Cantu RC (1998). Second-impact syndrome. Clin Sports Med.

[12] Saunders RL, Harbaugh RE (1984). The second impact in catastrophic contact-sports head trauma. JAMA.

[13] Tator CH (2013). Concussions and their consequences: current diagnosis, management and prevention. CMAJ.

[14] McCrory PR, Berkovic SF (1998). Second impact syndrome. Neurology.

[15] Cassidy JD, Cancelliere C, Carroll LJ, Côté P, Hincapié CA, Holm LW, Hartvigsen J, Donovan J, Nygren-de Boussard C, Kristman VL, Borg J (2014). Systematic review of self-reported prognosis in adults after mild traumatic brain injury: results of the International Collaboration on Mild Traumatic Brain Injury Prognosis. Arch Phys Med Rehabil.

[16] Stulemeijer M, van der Werf S, Borm GF, Vos PE (2008). Early prediction of favourable recovery 6 months after mild traumatic brain injury. J Neurol Neurosurg Psychiatry.

[17] Carroll LJ, Cassidy JD, Cancelliere C, Côté P, Hincapié CA, Kristman VL, Holm LW, Borg J, Nygren-de Boussard C, Hartvigsen J (2014). Systematic review of the prognosis after mild traumatic brain injury in adults: cognitive, psychiatric, and mortality outcomes: results of the International Collaboration on Mild Traumatic Brain Injury Prognosis. Arch Phys Med Rehabil.

[18] Cassidy JD, Boyle E, Carroll LJ (2014). Population-based, inception cohort study of the incidence, course, and prognosis of mild traumatic brain injury after motor vehicle collisions. Arch Phys Med Rehabil.

[19] Cassidy JD, Carroll L, Côté P, Holm L, Nygren A (2004). Mild traumatic brain injury after traffic collisions: a population-based inception cohort study. J Rehabil Med.

[20] Hartvigsen J, Boyle E, Cassidy JD, Carroll LJ (2014). Mild traumatic brain injury after motor vehicle collisions: what are the symptoms and who treats them? A population-based 1-year inception cohort study. Arch Phys Med Rehabil.

[21] Ferrari R, Russell AS, Carroll LJ, Cassidy JD (2005). A re-examination of the whiplash associated disorders (WAD) as a systemic illness. Ann Rhem Dis.

[22] Holm LW, Carroll LJ, Cassidy JD, Skillgate E, Ahlbom A (2008). Expectations for recovery important in the prognosis of whiplash injuries. PLoS Med.

[23] Carroll LJ, Holm LW, Ferrari R, Ozegovic D, Cassidy JD (2009). Recovery in whiplash-associated disorders: do you get what you expect?. J Rheumatol.

[24] Deyo RA, Diehl AK, Rosenthal M (1986). How many days of bed rest for acute low back pain? A randomized clinical trial. N Engl J Med.

[25] Silverberg ND, Iverson GL (2013). Is rest after concussion “the best medicine?” Recommendations for activity resumption following concussion in athletes, civilians, and military service members. J Head Trauma Rehabil.

[26] Nygren-de Boussard C, Holm LW, Cancelliere C, Godbolt AK, Boyle E, Stålnacke BM, Hincapié CA, Cassidy JD, Borg J (2014). Nonsurgical interventions after mild traumatic brain injury: a systematic review. Results of the International Collaboration on Mild Traumatic Brain Injury Prognosis. Arch Phys Med Rehabil.

[27] Chou R, Qaseem A, Snow V, Casey D, Cross JT, Shekelle P, Owens DK, Clinical Efficacy Assessment Subcommittee of the American College of Pain; American College of Physicians, American Pain Society Low Back Pain Guidelines Pain Guidelines Panel (2007). Diagnosis and treatment of low back pain: a joint clinical practice guideline from the American college of physicians and the American pain society. Ann Intern Med.

[28] Cancelliere C, Kristman VL, Cassidy JD, Hincapié CA, Côté P, Boyle E, Carroll LJ, Stålnacke BM, Nygren-de Boussard C, Borg J (2014). Systematic review of return to work after mild traumatic brain injury: results of the International Collaboration on Mild Traumatic Brain Injury Prognosis. Arch Phys Med Rehabil.

[29] Cancelliere C, Cassidy JD, Colantonio A, Loisel P, Anema JR (2013). Specific disorder-linked determinants: traumatic brain injury. Handbook of Work Disability: Prevention and Management.

[30] Hung R, Carroll LJ, Cancelliere C, Côté P, Rumney P, Keightley M, Donovan J, Stålnacke BM, Cassidy JD (2014). Systematic review of the clinical course, natural history, and prognosis for pediatric mild traumatic brain injury: results of the International Collaboration on Mild Traumatic Brain Injury Prognosis. Arch Phys Med Rehabil.

[31] Zemek R, Duval S, Dematteo C (2014). Guidelines for Diagnosing and Managing Pediatric Concussion [Internet].

[32] Godbolt AK, Cancelliere C, Hincapié CA, Marras C, Boyle E, Kristman VL, Coronado VG, Cassidy JD (2014). Systematic review of the risk of dementia and chronic cognitive impairment after mild traumatic brain injury: results of the International Collaboration on Mild Traumatic Brain Injury Prognosis. Arch Phys Med Rehabil.

[33] McKee AC, Cantu RC, Nowinski CJ, Hedley-Whyte ET, Gavett BE, Budson AE, Santini VE, Lee HS, Kubilus CA, Stern RA (2009). Chronic traumatic encephalopathy in athletes: progressive tauopathy after repetitive head injury. J Neuropathol Exp Neurol.

[34] Marras C, Hincapié CA, Kristman VL, Cancelliere C, Soklaridis S, Li A, Borg J, Af Geijerstam JL, Cassidy JD (2014). Systematic review of the risk of Parkinson’s disease after mild traumatic brain injury: results of the International Collaboration on Mild Traumatic Brain Injury Prognosis. Arch Phys Med Rehabil.

[35] Kristman VL, Borg J, Godbolt AK, Salmi LR, Cancelliere C, Carroll LJ, Holm LW, Nygren-de Boussard C, Hartvigsen J, Abara U, Donovan J, Cassidy JD (2014). Methodological issues and research recommendations for prognosis after mild traumatic brain injury: results of the International Collaboration on Mild Traumatic Brain Injury Prognosis. Arch Phys Med Rehabil.

